# “I Wonder What They *Do* Teach Them in These Schools”: *The Chronicles of Narnia* and Nature-Deficit Disorder

**DOI:** 10.1007/s10583-022-09515-7

**Published:** 2022-12-20

**Authors:** Sarah Selden

**Affiliations:** University of Oxford, Oxford, UK

**Keywords:** C.S. Lewis, Narnia, Nature-Deficit Disorder, Richard Louv

## Abstract

Throughout *The Chronicles of Narnia*, nature plays a prominent role in driving good’s triumph over evil, and while Lewis’s environmental activism in writing *Narnia* has gained critical attention, the connection between his penchant for nature and his educational philosophy has not yet been accordingly considered. Stemming from his early studies of Wordsworth’s *Prelude,* Lewis illustrates the crucial correlation between children spending time in nature and developing imagination. Lewis replaces English school systems and educational philosophy with natural images ranging from the transformation of pupil desks into rose bushes in *Prince Caspian* to Aslan’s emergence from the forest and subsequent abolition of Experiment House in *The Silver Chair*, connecting nature’s role in children’s moral development to their formal education. Lewis’s concerns expressed in *Narnia* draw renewed relevance with the prominence of technology and standardized testing in today’s classrooms. As Richard Louv argues in *Last Child in the Woods,* twenty-first century children are experiencing an alienation from nature that is causing physical and spiritual damage*.* This paper will explore Lewis’s educational philosophy as evidenced within the natural settings of *The Chronicles of Narnia,* its ramifications in a twenty-first century context and the real-world results of incorporating nature-based education into classrooms for all ages.

Nature-Deficit Disorder is a prominent issue among twenty-first century children who consistently trade time spent outdoors for time spent on screens. Even education systems are shortening recess time in exchange for seat time in hopes of producing better test scores. A 2016 study conducted by Great Britain’s National Trust found that twenty-first century children spend less than half the amount of time outdoors that their parents did at their age (Press Association, 2016). This trend is contributing to a growing pattern of nature-deficit disorder, a non-medical diagnosis coined by Richard Louv that consists of growing attention difficulties and irritability as well as physical and emotional illness. Despite its correlation with greater dependence on screens and standardized testing, nature-deficit disorder began affecting children long before the advent of personal devices. In the mid-twentieth century, C.S. Lewis, too, noted the correlation between spending time in nature and a child’s educational, emotional, and imaginative well-being. He connects these ideas in a range of his works, including his poetry, his spiritual autobiography *Surprised by Joy, (1955)* and his educational treatise *The Abolition of Man. (1943)* He most strongly develops this philosophy, though, across his children’s books *The Chronicles of Narnia,* as characters like Edmund Pevensie and Eustace Scrubb grow into better citizens in a way that directly correlates with the amount of time they spend outdoors. Throughout *Narnia,* Lewis replaces broken education systems with natural imagery, expressly arguing for the necessity of nature in “good” education systems. This marks Lewis as a progressive educational philosopher who would anticipate Richard Louv’s arguments nearly sixty years before nature-deficit disorder was named.

In his landmark book *Last Child in the Woods: Saving Our Children from Nature Deficit Disorder,* Louv asserts that while nature-deficit disorder is a non-medical diagnosis, its effects are nonetheless prominent in twenty-first century children: “Nature-deficit disorder describes the human costs of alienation from nature, among them: diminished use of the senses, attention difficulties, and higher rates of physical and emotional illness” (2008, p. 36). Louv’s theories were so widely read that he was awarded the Audubon Medal for his research efforts in 2008. He has continued to expand his research and recommendations over the past decade in subsequent books like *The Nature Principal* (2011), *Vitamin N: The Essential Guide to a Nature-Rich Life* (2016) and *Our Wild Calling: How Connecting with Animals Can Transform Our Lives—and Save Theirs* (2019).

Louv’s interest and concern with how much time children spend outside stems from the experiences of his own childhood. In his introduction*,* Louv remembers how “Americans around my age, baby boomers or older, enjoyed a kind of free, natural play that seems, in the era of kid pagers, instant messaging, and Nintendo, like a quaint artifact” (2008, p. 1). While this disconnect from nature may have begun much earlier, Louv is most concerned with children born in the 1980s, 90s, and 2000s, whose “society is teaching young people to avoid direct experience in nature” (p. 2), claiming that this lesson “is delivered in schools, families, even organizations devoted to the outdoors, and codified into the legal and regulatory structure of many of our communities” (p. 2). Even as teachers attempt to instill environmental stewardship in their students by teaching them about the plight of the rainforests or the importance of recycling, these lessons often unfortunately have the opposite effect if they are not coupled with adequate time spent outdoors— “lacking a direct experience with nature, children begin to associate it with fear and apocalypse, not joy and wonder” (p. 134). Louv suggests that developing this sense of joy and wonder associated with nature is essential for both proper child development and a proper pro-environmental attitude, and its absence from current educational practices will only become a more critical issue in the coming years as the effects of climate change become more pronounced.

Louv’s theories drew renewed relevance during the COVID-19 crisis, when parents began to notice negative behavioral changes in their children during stay-at-home orders. As Meg St-Esprit McKivigan reported in a June 2020 *New York Times* article, many parents watched their children become more “moody and cranky” after months of playground closures and staying inside due to overcrowded parks. McKivigan discussed this pattern with Louv, who said “ironically, the 2020 coronavirus pandemic, as tragic as it is, has dramatically increased public awareness of the deep human need for nature connection, and is adding a greater sense of urgency to the movement to connect children, families and communities to nature” (2020). Not only do children need to experience nature in order to be less “moody and cranky,” but nature also provides an excellent opportunity for moral education. In a chapter of *Last Child in the Woods* entitled “Telling Turtle Tales: Using Nature as a Moral Teacher,” Louv discusses a fishing trip he took with Robert F. Kennedy Jr. as part of his research. During that trip, Kennedy told him, “by polluting so that people can’t fish, or by making so many rules that people can’t get on the water, it’s the moral equivalent of tearing the last pages out of the last Bible on Earth” and that “our children ought to be out there on the water…this is what connects us, this is what connects humanity, this is what we have in common. It’s not the internet, it’s the oceans” (2008, p. 200). Nature is necessary for proper moral and behavioral development, and the educational upheaval caused by Covid-19 may be the opportunity educators and theorists need to reimagine how nature is incorporated into the educational experience.

Lewis strongly connects healthy moral development to a healthy connection to nature throughout the *Chronicles of Narnia,* but he is not the first British children’s writer to do so. One can trace this idea back to William Wordsworth’s poetry, which served as an important body of inspiration for Lewis. Other children’s writers throughout British history, noted the necessity of nature to a child’s “proper” upbringing. Tom Brown often turned to pony rides in the valley for solace from his hellish boarding school environment in *Tom Brown’s Schooldays,* and time spent outdoors in the garden directly correlates with healing and emotional growth in Frances Hodgson Burnett’s *The Secret Garden,* to name a few. It is only Lewis, though, who physically replaces schools like Experiment House in *The Silver Chair* with explicitly natural imagery, making nature’s connection to healthy child development even more urgent in the Narniad. This essay will explore both the development and ramifications of this idea. I will first evaluate relevant current scholarship regarding Lewis and nature before tracing Lewis’s love for nature back through his readings of Wordsworth’s *Prelude* and how it influenced his own imaginative and spiritual development in his childhood as he describes it in *Surprised by Joy.* I will then track how he develops these ideas both in his poetry and in *The Abolition of Man* before exploring how he more explicitly develops his theory of nature-based education through *The Chronicles of Narnia.* Finally, I will turn to real-word examples of educators implementing nature-based education programs in their schools in a variety of ways, from field trips to reading nature-rich books in class in spaces where direct access to nature may not be readily available. In undertaking this project, I will uncover the progressive educational theorist that C.S. Lewis was and seek to understand whether reading books like *Narnia* can truly help alleviate symptoms of nature-deficit disorder, in hopes of providing yet another strategy to help today’s children healthily cope with the ramifications of both the COVID-19 pandemic and living in a society where more and more time is spent indoors.

## C.S. Lewis as a Progressive Educator

Even in the 1950s, a time period in which many romanticized children spending more time outdoors, Lewis recognized an unfortunate disconnect between nature and children that results from the way they are educated—and he seeks to remedy this issue in *Narnia*. Early in *The Lion, the Witch, and the Wardrobe* (*LWW*)*,* Professor Kirk famously exclaims “I wonder what they *do* teach them in these schools” (1998a, p. 50) in response to Peter and Susan’s struggle to accept Lucy’s fanciful stories about her adventures in the wardrobe. While this may seem like a flippant rail against his contemporary education system, throughout the series Lewis develops a much more pointed critique of the way schools prevent children from developing imagination. To Lewis, imagination developed in nature is essential to a proper education, and he expresses this idea throughout *The Chronicles of Narnia, The Abolition of Man*, *Surprised by Joy,* and many of his letters.

Despite the natural imagery in his works and even the nature reserve named for him near his property in Oxford, Clare Etcherling argues that *Narnia* cannot be read ecocritically because “texts from the mid-twentieth century, such as *The Chronicles of Narnia,* are often extremely conservative and replicate imperial, racist values and ways of thinking from the nineteenth century” (2016, p. 96). Lewis is no doubt a conservative, religious thinker, and there is also little doubt that his ecophilia stems from his Christian faith. Even Etcherling admits that “indeed, his fiction is undeniably rife with detailed descriptions of fantastic landscapes and environments, and his characters often express a deep respect and admiration for the world around them” (p. 99). However, she also asserts that it is only the green, English countryside-like environment that Lewis values in his series:

It depicts English, pastoral environments in hierarchal and binary opposition to environments associated with non-white peoples and promotes a type of environmental stewardship based on his own interpretation of Christian theology that suggests only white, civilized, and Christian people are capable, rightful environmental stewards. As such, these are not untroubled environmental texts, as some critics seem to suggest, but a form of conservative pastoral literature that promotes supposedly universal attitudes to the environment—attitudes that are actually culturally specific and closely bound to both paternalistic and adventuresome imperial beliefs and behaviors. (p. 100)

Notably, Etcherling assumes that Lewis is drawing from the English countryside to describe Narnian landscapes, when in reality, his childhood spent living on a large estate in Belfast, Ireland ignited his initial love for nature. The fact that Etcherling takes issue with Lewis’s depiction of the Calormenes in Narnia is understandable, especially when reading *The Chronicles of Narnia* through a twenty-first century lens. However, these are not the only villains in Narnia to abuse nature, demonstrating that to deem Lewis’s environmental stewardship as unequivocally tied to his imperialistic views ignores his larger argument that the exploitation of nature is evil and damaging in general, no matter the perpetrator.

Ultimately, Lewis uses the villains in Narnia to emphasize the moral implications of how humans view and interact with nature. They model what he criticizes as what the “modern child wants” in his 1952 essay “On Three Ways of Writing for Children” (1982, p.45). Lewis discusses how a woman had sent him a manuscript for a children’s book in which the protagonist received a “wonderful gadget” from a fairy, “a thing of taps and handles and buttons you could press. You could press one and get an ice cream, another and get a live puppy” (p. 45). He told the writer of the manuscript that he “didn’t much care for that sort of thing,” to which the writer agreed that it “bores [her] to distraction, but it is what the modern child wants” (p. 45). In *The Magician’s Nephew (MN),* Uncle Andrew and Jadis (who later becomes the White Witch) view the magic fertility of Narnia’s land much like this “gadget”—it is something they can abuse for their own gain. In *Narnia and the Fields of Arbol: The Environmental Vision of C.S. Lewis,* Dickerson and O’Hara write that “Jadis and Uncle Andrew are so accustomed to abusing creation, and so used to the abundance of things, that they cannot appreciate the scene of creation” (2009, p. 95). Uncle Andrew instead focuses on the economic possibilities of the incredibly rich soil. After seeing the lamp-post spring up from the ground where Jadis threw the iron bar, he reveals his exploitative view of nature:

But what was America to this? The commercial possibilities of this country are unbounded. Bring a few old bits of scrap iron here, bury 'em, and up they come as brand new railway engines, battleships, anything you please. They'll cost nothing, and I can sell 'em at full prices in England. I shall be a millionaire. (Lewis 1998b, p. 127-8)

Andrew’s limited view of the beauty of nature is manifested in his lack of imagination as well—when the beasts talk, he hears only grunts, and believes until the moment he goes home that they are out to kill him. Jadis also misuses nature, eating the apple of the tree Diggory is told not to eat from for her own benefit rather than appreciating it for its power and later causing the unnatural long winter in *LWW*. Thus, Lewis does not only frame the Calormenes as people who cannot appreciate nature with the necessary wonder that both he and Louv view as paramount—the English Uncle Andrew and Charnian Jadis believe that nature is something to be dominated and exploited as well. To these “childish” adult characters, the magic of Narnia is nothing more than a “world of wish-fulfillment” (1982, p. 56), and so they cannot enjoy the personal growth-inducing benefits of spending time in such a rich, natural space.

Lewis’s respect for the natural world is evidenced in works published before *Narnia* as well. His 1938 poem “The Future of Forestry” demonstrates this attitude, as it advocates for the preservation of nature for the sake of its imaginative power. Lewis wrote this poem in response to the massive deforestation and then afforestation effort undertaken by the British government during and after World War I. While replacing the trees felled to fuel the British war effort may seem ultimately beneficial, in reality, the reasoning behind this project was purely militaristic—the British government did not want to be caught up in another war without the necessary lumber (Sheil, 2002, p. 82). Lewis balks at this exploitative view of nature and begins the poem asking the question, “How will the legend of the age of trees/ Feel, when the last tree falls in England?” (“The Future of Forestry, 1964, ll. 1–2). The poem continues with the voice of a child asking, “What was a chestnut?” (l.11) and “Say what it means to climb a beanstalk” (l.12). The speaker in the poem laments the loss of trees because of the sense of wonder they produce, describing them as “Trees as men walking, wood romances/ of goblins stalking in silky green” (ll. 21–22). Children living in a world without trees also live in a world without this imaginative imagery, and so Lewis values the forest not for its usefulness in war or even daily life but for its stimulating imaginative power. While nature-deficit disorder was not a named issue during Lewis’s lifetime, he clearly recognized the danger of children losing their connection with nature, and so he frames much of his literature with a pro-environmental message and demonstrates the necessity of nature’s presence in the education system.

Etcherling’s analysis of Lewis’s environmental attitude also overlooks his relationship with William Wordsworth’s poetry. Lewis’s appreciation for the poet and how he shapes his view of nature’s role in education is well established in *Surprised by Joy* and many of his letters. Nature acts as teacher for both Wordsworth and Lewis, and this idea elucidates how nature functions in Narnia. The *Prelude* is perhaps the most influential of Wordsworth’s works in Lewis’s life—in a letter to Dom Bede, Lewis writes “The *Prelude* has accompanied me through all the stages of my pilgrimage: it and *Aeneid* (which I have never felt you value sufficiently) are the two long poems to wh. [sic] I most often return” (*C.S. Lewis Collected Letters, Vol. II*, 2004, p. 111). Both Wordsworth and Lewis discredited their respective contemporary education systems because of the way they prevented children from experiencing the wonder of nature, and Wordsworth expresses this idea in *Prelude*. Wordsworth believed that his contemporary schools, which consisted of “book learning and accumulation of fact” (Gill, 1991, p. 66), produce children whose “discourse moves slow/ Massy and ponderous as a prison door…the path in which he treads/ is choked with grammars” (1979, ll. 311–12, 15–16). Louv, Lewis, and Wordsworth believe that children educated in this way are stripped of imaginative development, a process linked to time spent both in nature and books for the latter two writers. In a section of the *Prelude* discussing coming home from school for the holidays, Wordsworth likens the imaginative joy he experiences while reading *Arabian Nights* to time spent outdoors:

Returning at the holidays, I found

That golden store of books which I had left

Open to my enjoyment once again,

What heart was mine! Full often through the course

of those glad respites in the summertime

When armed with rod and line we went abroad

For a whole day together, I have lain

Down by the side, O Derwent, murmuring stream,

On the hot stones and in the glaring sun (1979, ll. 79–85).

Wordsworth biographer Stephen Gill writes that, in this passage, the poet “urges freedom, the freedom of the boy who hoots to owls across Windermere, the freedom of imagination to lose itself in *The Arabian Nights*, the freedom to learn by indirection and delight” (Gill, 1991, p. 66). He continues, “Book V reaffirms the primacy of Imagination, grounded in Nature, fostered and strengthened by books. And the process of growing, *The Prelude* asserts, must be done in that order” (p. 66). To Wordsworth, learning fostered by both nature and imagination is essential for proper human development—this is what prevents children from developing what we today may call nature-deficit disorder. It is only when young Wordsworth is at home, away from the sterile education system, that this process can take place.

Lewis expresses similar views in his own autobiographical work, *Surprised by Joy.* While his parents “cared for no poetry at all” (Lewis, 2012, p. 4), he nonetheless calls himself a “romantic” (p. 3). As a child in Belfast, he often played in the back garden, and this was essential to developing his imagination. Early in his autobiography*,* he remembers a toy garden that his brother made out of moss and twigs as something that made him “aware of nature—not, indeed, a storehouse of forms and colours but as something cool, dewy, fresh, exuberant” (p. 6). Lewis was also a voracious reader as a child, and just as Wordsworth links reading *Arabian Nights* to sitting outside by the stream in *Prelude,* Lewis connects reading to nature, claiming that he “had the same certainty of finding a book that was new to me as a man who walks into a field has of finding a new blade of grass” (p. 10). Lewis then shares three of his own imaginative episodes that he claims defined his life, allowing him to develop a sense of wonder about nature that he believes is essential for proper imaginative development. He writes, “the reader who finds these three episodes of no interest need read this book no further, for in a sense the central story of my life is about nothing else” (p. 19), further emphasizing their importance in both his personal and educational philosophy. The first episode is the “memory of a memory” of his brother’s toy garden, and the second is a glimpse that came through reading *The Tale of Squirrel Nutkin,* which he says

…troubled me with what I can only describe as the Idea of Autumn. It sounds fantastic to say that one can be enamoured of a season, but that is something like what happened; and as before, the experience was one of intense desire. And one went back to the book, not to gratify the desire (that was impossible—how can one *possess* Autumn?) but to reawake it. (p. 18)

It is important to note that this imaginative experience comes from reading a book laden with natural imagery, much as his own children’s books would cultivate for other children in the future. The third imaginative experience comes from reading Longfellow’s *Tegner’s Drapa,* which Lewis says made him feel “instantly uplifted into huge regions of northern sky” (p. 18) and desire “with almost sickening intensity something never to be described… and then, as in the other examples, found myself at the very same moment already falling out of that desire and wishing I was back in it” (p. 18). This desire echoes Wordsworth’s “visionary gleam” that he famously describes in “Ode: Intimations of Immortality from Recollections of Early Childhood,” and Lewis indeed calls this desire a “visionary gleam” (p. 291) in the final pages of *Surprised by Joy*. For both writers, nature, imagination, and reading work together to produce this visionary gleam, or the sense of wonder that Lewis attaches to nature in *Narnia.* It is also the same sense of wonder for which Louv advocates in *Last Child in the Woods.* This imaginative wonder is, as all three writers argue, essential to a child’s proper education, transforming young learners into good citizens that help move society forward.

## Imaginative, Nature-Based Education in *Narnia*

Lewis further links nature to a proper, imaginative education in both *Narnia* and *The Abolition of Man*. Imagination is necessary to enjoy *Narnia* fully, as Lewis notes in the dedication of *LWW.* He tells Lucy Barfield that at the time of the book’s publication, she is too old for fairy tales, but that “some day you will be old enough to start reading fairy tales again” (Lewis, 1998a, p. 4). This cultivation of imagination is integral to Lewis’s philosophy of education, regardless of the character or reader’s stage of life. However, Lewis’s contemporary education system, like Wordsworth’s, was not at all concerned with instilling imaginative wonder in its students. In *The Abolition of Man* (1946, p. 14), Lewis writes that “the task of the modern educator is not to cut down jungles but to irrigate deserts” because educators of his time “see the world around them swayed by emotional propaganda—they have learned from tradition that youth is sentimental—and they conclude that the best thing they can do is fortify the minds of young people against emotion” (p. 14). Just as Wordsworth likens his contemporary education system to “a prison door,” Lewis sees his as producing emotionless, desert-like minds in need of imaginative irrigation. Imagination was not properly valued in schools in either Wordsworth's or Lewis’s day, an idea especially problematic to Lewis because he believed that, as Joel Heck (2006, p. 28) argues*,* that while “the *task* of education is to irrigate deserts, the *purpose* of education is to produce a good citizen.” Lewis literalizes this metaphor in Shasta’s transformation in *The Horse and His Boy,* when Shasta must escape a life of indentured servitude in the deserts of Calormen to become a good, just king among the green hills of Archenland. As Lewis demonstrates throughout *Narnia,* it is the cultivation of imagination in nature that produces these good citizens.

From Professor Kirk’s first exclamation of “Why don’t they teach logic in these schools?” (Lewis, 1998a, p. 48) in *LWW*, Lewis establishes that children who have been educated in “our world” need to be re-educated in Narnia, and his demonstration of this idea intensifies as he writes the Narniad. From the outset, *Narnia* is a story of children leaving their urban, war-torn home to find the safety necessary to learn and play in the midst of WWII. They are sent to live in the countryside with a professor, who enables Lucy to find Narnia initially by providing the magical wardrobe, and later persuades Susan and Peter to believe Lucy’s stories of Narnia and eventually enter the world themselves. Lucy, Peter, and Susan are all more or less willing to accept what they see and learn from Tumnus and the Beavers and display a strong sense of wonder as they see Narnia for the first time: Peter’s enthusiasm to “go explore the wood, of course” (p. 55) leads his siblings to begin their Narnian adventure with a sense of great pleasure. However, Edmund’s selfish and jealous attitude is in need of acute transformation, and through Aslan’s rescue and ultimate sacrifice, Edmund’s heart changes for the better as the long Narnian winter melts.

In *LWW,* the Pevensie children grow up in Narnia and exit the Wardrobe as changed people, but it is in *Prince Caspian* (*PC*) where Lewis truly marks their time in Narnia as a rejection of their formal education, demonstrating that it is not only the “bad” children (like Edmund once was) that need to be re-educated in nature, but also the generally good children like Peter and Lucy. In *PC,* all four Pevensies are drawn into Narnia just before catching the train to go to back school after the summer holidays, explicitly interrupting their British formal educational experience. Once they arrive in Narnia, they spend time traveling through the woods (which the ruling Telmarines are too afraid to enter) to meet the rebels at Aslan’s Howe. In *Planet Narnia,* Michael Ward notes that in *PC,* Peter and Edmund do “not simply harden, [but] become knightly” (Ward, 2008, p. 89), and their time spent in nature is integral to this transformation. While spending time in “our world” in between the events of *LWW* and *PC* has somewhat transformed the Pevensies back into their former, childish selves, reentering Narnia as “the High King and his consorts” (Lewis, 1998c, p. 164) and spending time amongst the magical and imaginative woods helps them to regain their chivalrous qualities that they learned during their reign as kings and queens. At the end of *PC,* Peter tells Edmund and Lucy about a conversation he and Susan had with Aslan that morning in which Aslan informed them that the two older Pevensies would not return to Narnia. Lucy then says, “What awful bad luck. Can you bear it?” to which Peter responds with “Well, I think I can…It’s all rather different from what I thought. You’ll understand when it comes to your last time” (p. 207). Peter’s mature response to the news and the understanding he develops demonstrates that his and Susan’s re-education is complete—they are now charged to permanently leave Narnia and live as good citizens in our world. While it seems Peter was more successful at this than Susan, who eventually stops believing in Narnia, it is clear from this episode that the children of our world’s time in Narnia has a specific purpose, and once that purpose has been fulfilled, their visits come to an end. This idea, too, models what Lewis designates as the true benefit of reading fairy tales as children in “On Three Ways of Writing for Children”—such reading “stirs and troubles him (to his life-long enrichment) with the dim sense of something beyond his reach and, far from dulling or emptying the actual world, gives it a new dimension of depth” (1982, p. 57). Lewis also writes that the child reading fairy tales “does not despise real woods because he has read of enchanted woods: the reading makes all woods a little enchanted” (1982, p. 57). For the Pevensies, spending time in Narnia re-enchants the life they live in their own world, as they can now live life awoken to this sense of “longing,” or “visionary gleam” (*Surprised By Joy* p. 291) that Lewis believes is essential to proper moral development.

Before they leave Narnia at the end of *PC*, though, Lewis explicitly ties this new moral development to a replacement of their previous education in our world, as Susan and Lucy romp through Narnia with Aslan to physically tear down and replace the Telmarine English-style school system. Since the children left Narnia, the Telmarines (who originate in our world) have invaded and taken over, converting Narnia into something very similar to England: they have built bridges over the rivers (imprisoning the river spirits), pushed the “old Narnians” (the talking beasts, dwarves, fauns, etc.) into hiding, and set up English-style schools for human children to attend. As Aslan, Susan and Lucy, and a host of “old Narnians” run through Narnia to free her from Telmarine rule, they come upon a girls' school, “where a lot of Narnian girls, with their hair done very tight and ugly tight collars round their necks and thick tickly stockings on their legs were having a history lesson” (Lewis, 1998c, p. 186). Lewis’s narrator then tells the reader that “the sort of ‘History’ that was taught in Narnia under Miraz’s rule was duller than the truest history you ever read and less true than the most exciting adventure story” (p. 186).

This type of education directly corresponds with the system Lewis criticizes in *Abolition of Man—*it is striving to cut down the jungles in their mind rather than irrigate the deserts. It is not cultivating any moral values within the children because it is not true, nor is it fostering their imagination, and their constraining uniforms and strict desk learning mimic the type of data-driven education that Louv criticizes in *Last Child in the Woods*. As she is experiencing this type of education, Gwendolyn, a Narnian girl, peers out the window and sees Aslan staring through it. She tells the teacher, Miss Prizzle, who immediately tells her to “take two order-marks for talking nonsense” (Lewis, 1998c, p. 187) even in the magical land of Narnia. Aslan responds to this with a roar and dismantles the school entirely:

Ivy came curling up in at the windows of the classroom. The walls became a mass of shimmering green, and leafy branches arched overhead where the ceiling had been. Miss Prizzle found she was standing on grass in a forest glade. She clutched at her desk to steady herself, and found that the desk was a rose-bush. Wild people such as she had never even imagined were crowding round her. Then she saw the Lion, screamed and fled. (p. 187)

Aslan quite literally replaces the Telmarine institution of education with nature, indicating that it is among green grass and rosebushes where children can truly be educated. All the girls in the school flee except for Gwendolyn, who is freed from her uncomfortable school clothes and allowed to join the romp. As Aslan’s party travels through the town of Beruna, more symbols of this dry “education” system are converted into natural imagery: a boy being whipped finds that his oppressor has been turned into a tree, and a flock of unruly, piggish schoolboys is turned into actual pigs. As to not limit the availability of a natural, true education to only imaginative children, Aslan also frees a teacher from her duties at the boys' school just as he freed Gwendolyn, because she too saw him through the window and had enough imaginative power to understand the true nature of the “divine revelers” (p. 188). The education system in Beruna has been entirely replaced with natural imagery, and Gwendolyn and the schoolmistress are now free to learn imaginatively as they follow Aslan in his run through the green environment of Narnia.

When Lucy and Edmund next enter Narnia in *The Voyage of the Dawn Treader* (*VDT*)*,* they arrive prepared to experience all of nature’s joys, even as the magic picture transports them directly into the Narnian ocean rather than dry land. Despite being cold and wet, Lucy feels “quite sure they were in for a lovely time” (1998e, p. 17) as soon as she finds herself on board the *Dawn Treader.* In this chronicle, then, it is Eustace who needs to be transformed through an experience in the natural world, as his first reaction to the Narnian world is to yell out “Let me go. Let me go back. I don’t *like* it” (p. 12) after Caspian rescues him from the sea. At the beginning of *VDT*, Eustace is a perfect product of his English school system—a “victim of his education” (Ford, 1994, p. 306), and also almost a perfect example of a child experiencing nature-deficit disorder. He has no imaginative ability, and only finds interest in “animals, especially beetles, if they were dead and pinned on a card” (Lewis, 1998e, p. 11), and enjoys reading only if his materials “were books of information and had pictures of grain elevators or of fat foreign children doing exercises in model schools” (p. 11). Eustace also has an affinity for technology, proclaiming his admiration for the state-of-the-art *Queen Mary,* which had a “saloon,” “radio,” and “deck chairs” (p. 35) and has contempt for more “primitive” (p. 35) ships like the *Dawn Treader* that leave passengers exposed to the elements. Lewis’s view of modern technology was generally negative—to him, technology “refers to the increasing separation of humanity from nature brought on…by the application of the scientific method to all aspects of human life” (Ford, 1994, p. 409). Eustace is a prime example of a person affected by this mindset and thus desperately needs to be re-educated in Narnia.

In *Last Child in the Woods,* Louv describes the deep issues that come from a technology-laden world that today’s children grow up in, but Lewis noted these problems even before the invention of personal devices and saw a way to rectify it. Eustace’s transformation stems from his exposure to nature on the *Dawn Treader* and his brief imprisonment in the body of a dragon, during which most of his nights are spent “curled up like a snake between the wood and the water” (Lewis, 1998e, p. 98) thinking about his actions and treatment of others. This is then followed by his own encounter with Aslan in the middle of a garden (similar to Lewis’s own childhood garden) “with trees and fruit and everything” (p. 102) before he is finally released from his dragon state. After Eustace rejoins the *Dawn Treader* party, the narrator remarks that “it would be nice, and fairly true, to say that ‘from that time forth Eustace was a different boy.” To be strictly accurate, he began to be a different boy. He had relapses…but the cure had begun” (p. 106). His experience on a boat devoid of technology, at the mercy of the natural sea and wind, has prepared him to be transformed by Aslan by cultivating the imagination necessary to learn from this experience. Eustace then apologizes to his companions and develops a new appreciation for both them and the environment they continue to explore. By the end of the chronicle, people “back in our own world soon started saying how Eustace had improved, and how ‘you’d never know him for the same boy’” (p. 223), demonstrating just how drastically his Narnian re-education has transformed him for the better.

After Eustace’s transformation in *VDT,* Aslan uses him and Jill to dismantle another example of Lewis’s contemporary schools in *The Silver Chair* (SC), Experiment House, which is an example of what “progressive” schools were like during Lewis’s time (Ford, 1994, p. 363). At the end of *SC,* Jill, Eustace, and Caspian come bounding in from the woods of Aslan’s Country, a natural environment, as Aslan roars and breaks down the wall that was barring the school from the wood. He then reveals himself to the bullies at Experiment House, providing them with the imaginative image of “a lion as large as a young elephant lying in the gap, and three figures in glittering clothes with weapons in their hands rushing down upon them” (Lewis, 1998d, pp. 219–220). The bullies, who are also apt examples of children affected by nature-deficit disorder because of their education at Experiment House, cannot process what is happening to them and begin “running around like mad, crying out, ‘Murder! Fascists! Lions! It isn’t *fair!*”’(p. 220). Just as he destroyed the English-style Narnian school in *Prince Caspian*, Aslan destroys the negative parts of Experiment House through nature and imagination. After this task is complete, “things changed for the better at Experiment House, and it became quite a good school” (p. 221). To Lewis, children cannot be properly educated without developing an appreciation for nature, and his disbandment of his “traditional” contemporary education systems marks him as a progressive, pro-environmental educator.

Near the end of the Narniad writing process, when Lewis returns to the magical world’s origin story, he establishes the centrality of nature in Narnia’s ethos. In Narnia, it is an appreciation for ecology—not a formal education—that qualifies people to rule the country from the time of its creation story in *MN*. In this chronicle, Aslan clearly puts man in charge of creatures and nature in Narnia, instructing Frank the Cabby and his wife, Nellie, to “rule and name all these creatures, and do justice among them, and protect them from their enemies when enemies arise” (Lewis, 1998b, p. 158). Frank is given kingship of Narnia despite not having an “eddycation” (p.158)—he has not been spoiled by the English school system, and Aslan tells him all he needs to know to be a successful king is how to “use a spade and a plough and raise food out of the earth” (p. 158). Because of their lack of sterile, prison-door-like education, Frank and Nellie can both believe and appreciate the Narnian creation and have already developed the values needed to rule the country justly. As Lewis increases the bounds of his (and his reader’s) knowledge about Narnia by writing this prequel as the sixth story, he reveals one of the series’ core purposes: to reignite the connection between nature, imagination, and a proper education for both his characters and his readers. Frank and Nellie are two of the only truly “good” human adults readers meet in Narnia, and here Lewis explicitly demonstrates that it is their love of nature and open imagination that makes them so. This encourages Lewis’s readers to perhaps reevaluate their own relationship with nature just before reading *The Last Battle* and leaving Narnia for the last time.

## Imaginative, Nature-Based Education in Our World

Since Louv’s release of *Last Child in the Woods* in 2008, educators have adopted nature-infused educational philosophies across the United States that reflect the same ideas that Lewis develops in Narnia*.* Louv recounts many examples of real schools in our world finding success in implementing more nature-based, or at least nature-appreciative, educational programs similar to what Wordsworth and Lewis advocate for in their literature. He discusses the story of one secondary English teacher in Monterey, California, who took her students on a field trip to Monterey Bay while teaching Steinbeck’s *Cannery Row* so that they could see the tide pools described in the novel in person. Marine biologists led the trip, and Louv reports that “in addition to helping the students learn about natural science, Dankert's students discussed the meaning of community—because one of Steinbeck's characters had described a tide pool as a metaphor for the community of life…the trip helped the class form its own community” (Louv, 2008, p. 208). Just as characters in Narnia leave the magical, nature-laden realm as changed, better citizens, students in this class left their field trip as better citizens of their own community—they developed a better appreciation for their surroundings and for each other through this outdoor educational experience.

Just as his time on the *Dawn Treader* helped to rectify many of Eustace’s behavioral issues, time spent in nature can translate to fewer behavioral issues in real-world schools as well. Louv reports that Little Falls High School in Minnesota claims such success after implementing an environment-based program for their freshmen class, as they “reported that students in the environment-based program had 54 percent fewer suspensions than other ninth-graders” (2008, p. 206). Teachers and administrators also noted that spending more time outdoors led to greater educational engagement among the students, fulfilling an important goal in today’s educational practices. While Little Falls High School is located in a more suburban community, Louv argues that increased time outdoors can and must occur in urban neighborhoods as well. He writes that “today, a growing number of ecologists and ethicists are challenging the assumption that cities have no room for wildlife” (p. 246). In order to fully challenge the prevalence of nature-deficit disorder in twenty-first century children, urban ideals must be reframed. Louv posits that:

a healthy urban environment requires natural corridors for movement and genetic diversity. One can imagine such a theory applied to entire urban regions, with natural corridors for wildlife extending deep into urban territory and the urban psyche, creating an entirely different environment in which children would grow up and adults could grow old—where nature deficit is replaced by natural abundance. (p. 247)

While Louv concedes that many people would feel uncomfortable with sharing their morning commute with wildlife, he reminds readers that “a de-natured urban or suburban environment is not good for children or the land” (p. 249). Louv is not arguing that urban planners need to destroy bridges like Aslan does in *PC,* but in order to save children from the damaging effects of nature-deficit disorder, urban and suburban citizens must become comfortable with a more symbiotic relationship with their natural surroundings.

While nothing can replace time spent directly in nature, new research suggests that vicarious experiences—such as encountering nature through reading—can help alleviate symptoms of nature-deficit disorder as well. Stephen R. Kellert, the Tweedy Ordway Professor Emeritus of Social Ecology at Yale (2002, p. 135), writes that “vicarious experiences of the natural world help the young person to navigate the perilous issues of maturation in a vivid, often mysterious, and unusually beguiling way,” and that “when coupled with direct contact and immersion in nearby nature, these symbolic encounters provide extraordinary opportunities for psychosocial growth and development” (p. 135). While the results of vicarious experiences with nature may seem difficult to gauge, a group of researchers conducted such a study in Tokyo, measuring children’s response to nature and propensity to protect it after experiencing it both directly (going outside to experience the biodiversity of their neighborhood) and indirectly (through reading, viewing photographs, or watching documentaries). They found that

in the present study, the frequency of vicarious experiences of nature, as well as that of direct experiences, was positively associated with children’s affective attitudes toward local biodiversity. This result suggests that, in circumstances where a given amount of natural environment cannot immediately be preserved within the home ranges of children, vicarious experiences could, to some extent, be used as a subsidiary tool in attracting their interest in and care for biodiversity. (Soga et al., 2016)

It is important to note that this study was conducted in the heavily urban environment of Tokyo, proving that even children who grow up surrounded by concrete instead of forests can develop a positive and productive view of nature. Additionally, the children in this study did not associate nature with “fear and apocalypse” (Louv, 2008, p. 134) as Louv suggested because their vicarious encounters occurred in tandem with direct experiences. Lewis benefited from this growth in his own childhood through both playing in his garden and reading *Squirrel Nutkin,* developing the sense of imaginative wonder that Louv prescribes as necessary for a healthy childhood. Reading books like *Narnia,* which are so full of natural imagery and respect thereof, can also lead to the development of an appreciation of nature, not in the dry, scientific way that schools often teach, but in a manner that allows children to exert their imagination and avoid the symptoms of nature-deficit disorder.

This idea that both direct and vicarious experiences with nature are necessary to a proper education has greater implications than even ensuring healthy child development. Louv writes that “reducing that deficit—healing the broken bond between our young and nature—is in our self-interest, not only because aesthetics or justice demands it…the health of the earth is at stake as well” (2008, p. 6). While we can (and should) continue to search for alternative energy sources and seek to reduce waste, Louv believes that the only way to truly solve the current global climate crisis is to instill a wonder-producing love of nature in our children. He continues, “how the young respond to nature, and how they raise their own children, will shape the configurations and conditions of our cities, homes—our daily lives” (p. 6). *Narnia* is of course not the only children’s series that can provide children with vicarious natural experiences, but it provides ample examples of children transformed by green, imaginative learning that irrigates the deserts of their minds and replaces the dry, sterile education they have received thus far. Louv, Lewis, and Wordsworth all provide a progressive window to what good, nature-infused education can be, and if twenty-first century educators can implement the ideals they prescribe, the next generation of students will not only be better behaved, more well-rounded, and healthier overall, but they will also be better environmental stewards ready to fully address our global climate crisis.
